# Magnitude of postpartum hemorrhage and its associated factors in Ethiopia: a systematic review and meta-analysis

**DOI:** 10.1186/s12978-022-01360-7

**Published:** 2022-03-09

**Authors:** Jemberu Nigussie, Bekahegn Girma, Alemayehu Molla, Takla Tamir, Ruth Tilahun

**Affiliations:** 1 Department of Nursing College of Health Sciences and Medicine, Dilla University, Dilla, Ethiopia; 2 Department of Psychiatry College of Health Science and Medicine, Dilla University, Dilla, Ethiopia; 3 Department of Midwifery, College of Health Science and Medicine, Dilla University, Dilla, Ethiopia

**Keywords:** Postpartum hemorrhage, Bleeding after birth, Obstetric complications, Magnitude, Prevalence, Ethiopia

## Abstract

**Background:**

Postpartum hemorrhage or postpartum bleeding (PPH) is often defined as loss of > 500 ml of blood after vaginal delivery or > 1000 ml after cesarean delivery within 24 h. Postpartum hemorrhage is a leading direct cause of maternal morbidity and mortality in Ethiopia. Therefore, the main objective of this systematic review and meta-analysis was to estimate the pooled magnitude of postpartum hemorrhage and the pooled effect size of the associated factors in Ethiopia.

**Methods:**

Primary studies were searched from PubMed/MEDLINE online, Science Direct, Hinari, Cochrane Library, CINAHL, African Journals Online, Google and Google Scholars databases. The searching of the primary studies included for this systematic review and meta-analysis was limited by papers published from 2010 to October 10/2021. The data extraction format was prepared in Microsoft Excel and extracted data was exported to Stata Version 16.0 statistical software for analysis**.** A random effect meta-analysis model was used. Statistical heterogeneity was evaluated by the I^2^ test and Egger’s weighted regression test was used to assess publication bias.

**Result:**

A total of 21 studies were included in this meta-analysis. The pooled magnitude of postpartum hemorrhage in Ethiopia was 8.24% [(95% CI 7.07, 9.40]. Older age [OR = 5.038 (95% CI 2.774, 9.151)], prolonged labor [OR = 4.054 (95% CI 1.484, 11.074)], absence of anti-natal care visits (ANC) [OR = 13.84 (95% CI 5.57, 34.346)], grand-multiparty [OR = 6.584 (95% CI 1.902, 22.795)], and history of postpartum hemorrhage [OR = 4.355 (95% CI 2.347, 8.079)] were factors associated with the occurrence of postpartum hemorrhage.

**Conclusions:**

The pooled magnitude of postpartum hemorrhage among post-natal mothers in Ethiopia was moderately high. The finding of this study will strongly help different stakeholder working in maternal and child health to focus on the main contributors’ factors to reduce post-partum hemorrhage among postnatal mothers. Health professionals attending labor and delivery should give more attention to advanced aged mothers, grand-multipara mothers and mothers who had a history of post-partum hemorrhage due to higher risk for postpartum hemorrhage. Encouraging to continue ANC visit and prevent prolonged labor should also be recommended to decrease postpartum hemorrhage.

**Supplementary Information:**

The online version contains supplementary material available at 10.1186/s12978-022-01360-7.

## Plain Language summary

Even though other complications occur during pregnancy, post-partum hemorrhage (PPH) is the most serious complication and most critically important cause during pregnancy and child birth. It can cause severe anemia, acute respiratory distress syndrome (ARDS), acute renal failure (ARF), coma, and cardiac arrest. Uterine atony, retained tissue, genital tract tear, coagulation problem, and uterine rupture are most common causes of post-partum hemorrhage. In developing countries, PPH is one of the leading causes of maternal mortality, accounting for 25–43% of maternal death. It is also a leading cause of maternal morbidity and mortality in Ethiopia. This systematic review and meta-analysis were performed following the preferred reporting items for systematic reviews and Meta-Analyses (PRISMA). Primary studies were searched from different databases and random effect meta-analysis model was used. This systematic review and meta-analysis included 21 primary studies with a total of 93,898 study participants. The pooled magnitude of postpartum hemorrhage in Ethiopia was 8.24%. Advanced maternal age, prolonged labor, absence of antenatal care (ANC) visits, grand multi-parity, previous history of postpartum hemorrhage were significantly associated with postpartum hemorrhage. The finding of this systematic review and meta-analysis will strongly help different stakeholders working in maternal and child health to focus on the main contributor factors to reduce PPH If postpartum hemorrhage is reduced, it is the fact that maternal death will be decreased.

**Supplementary Information:**

The online version contains supplementary material available at 10.1186/s12978-022-01360-7.

## Introduction

Postpartum hemorrhage or postpartum bleeding (PPH) is often defined as loss of > 500 ml of blood after vaginal delivery or > 1000 ml after cesarean delivery within 24 h [1–4]. It is also defined as blood loss sufficient to cause hypovolemia, a 10% decrease in hematocrit or requiring transfusion of blood products regardless of the route of delivery [1, 5, 6].

Postpartum hemorrhage (PPH) is the most common complication of deliveries and its magnitude is reported to be 2–4% and 6% after vaginal and cesarean-sections (C/S) deliveries respectively [7, 8]. Uterine atony is responsible for more than 50% of PPH cases, followed by retained tissue, genital tract tear, coagulation problem, and uterine rupture [2, 8]. PPH has long and short term impacts like chronic illness, disability, increased risk of death and/or poor growth and development of their children, hepatic dysfunction, adult respiratory distress syndrome and renal failure [9–12].

PPH occurs approximately 8.7 million times and causes 44,000 to 86,000 deaths per year, making it the leading cause of death during pregnancy globally [13, 14]. In developing countries, PPH is the leading cause of maternal death, accounting for 25–43% of maternal death, unlike those of developed countries in which pulmonary embolism is the leading cause of maternal mortality [7]. Postpartum hemorrhage is a serious problem even in metropolitan areas of sub-Saharan Africa [15].

Ethiopia is one of the countries with the highest maternal mortality rate (MMR) and almost all of these deaths were due to direct obstetric complications [16]. PPH is a leading direct causes of maternal morbidity and mortality in all region of the country [16]*.* A study conducted in Jima revealed that 54% of maternal deaths were caused by postpartum hemorrhage [10]. Similarly, 46.5% of maternal mortality in the kersa district was due to post-partum hemorrhage [17].

Risk factors for PPH includes; past history of PPH, multiple pregnancy, fetal macrosomia, prim-gravidity, grand multi-parity, older age, preterm births, genital tract injuries, non-use of oxytocic’s for PPH prophylaxis, absence of anti-natal care (ANC), labor induction, duration of labor, cesarean birth and intra-uterine fetal deaths [12, 18–22].

Postpartum hemorrhage is the most preventable and treatable problem through active management of the third stage of labor (AMTSL) [23]*.* However, the use of oxytocin is not feasible in many low-income countries, where most births take place at home with untrained birth attendants [22, 24]. The preference of mothers to deliver at home rather than in health facility is the main cause of maternal mortality in Ethiopia [25].

Even though the maternal death is decreased in Ethiopia, it is still high according to WHO maternal death classification [16]. Postpartum hemorrhage (54%) is the leading cause of maternal death in the country [10]. The government of Ethiopia launches different strategies to prevent postpartum hemorrhage while maternal mortality due to hemorrhage is still high [25]. This is due to low utilization of existing maternal health services, poor access and unavailability of quality obstetric care in most of health facilities, so decreasing PPH and related maternal death still remained challenge in Ethiopia [26–28].

In Ethiopia, the magnitude of postpartum hemorrhage was varied from region to region and the associated factors identified by the primary studies were inconsistent. The magnitude of postpartum hemorrhage in Addis Ababa was 1.4% [29] but 16.6% in South Nation Nationality People region [30]. Furthermore, there is no nationally representative pooled data on the magnitude of postpartum hemorrhage in Ethiopia. Therefore, reliable and summarized information is essential to refine the government policies, strategies, and interventions. The main objective of this systematic review and meta-analysis was to estimate the pooled magnitude of postpartum hemorrhage and the pooled effect size of associated factors in Ethiopia.

### Research questions

What is the pooled magnitude of postpartum hemorrhage among post-natal mothers in Ethiopia?

What is the pooled effect size of factors associated with postpartum hemorrhage among post-natal mothers in Ethiopia?

## Methods

### Eligibility criteria

This review includes observational studies (cross-sectional, case–control, cohort and survey) these reported the magnitude of postpartum hemorrhage among postnatal mothers in Ethiopia published from 2010 to 2021. The last search date for this study is October 10/2021. We include articles published in the English language. All studies were conducted in either health institutions or communities were included. However, case reports, qualitative studies, and articles without full text were not included in this systematic review and meta-analysis.

### Information source

This systematic review and meta-analysis was performed in accordance with the preferred reporting items for systematic reviews and Meta-Analyses (PRISMA) guideline [31, 32] (Additional file 1). These published and unpublished (Grey literature) researches report the magnitude or prevalence of postpartum hemorrhage and associated factors in Ethiopia were included in this review.

### Search strategies

Primary studies were searched from PubMed/MEDLINE online, Science Direct, Hinari, Cochrane Library, CINAHL and African Online Journals databases. Grey literature was also identified from Google and Google Scholars. We have searched using controlled vocabulary variables such as postpartum hemorrhage, postpartum bleeding, maternal bleeding and birth outcome and birth complications. We used key terms to retrieve primary studies (magnitude OR prevalence AND postpartum hemorrhage OR bleeding AND Ethiopia. For factors associated with post-partum hemorrhage; factors OR determinants OR risk factors OR correlations AND postpartum hemorrhage key terms were used. Two authors (JN and BG) searched the primary studies from different databases.

### Study selection and quality appraisal

The principal investigator (JN) performed an initial review by title and abstract to eliminate articles that were visibly not important to this review. The full text articles were included if they reported the magnitude of postpartum hemorrhage and/or its associated factors. Two reviewers (BG and AM) independently screened the selected studies using pre-specified inclusion criteria. During the selection process, disagreements between the two authors were decided by the mediation of other reviewers (TT, RT).

The authors used the Newcastle–Ottawa quality assessment scale to assess the qualities of the included studies [33]. The tool has three main parts. The first five components assess the methodological quality of each study. The second part assesses the comparability of primary studies, and the final part of the tool measures the quality of the original articles with respect to their outcome and statistical analysis. All articles scored 7 and more can be considered as low risk and good to be included for the meta-analysis.

### Outcome of measurements

This systematic review and meta-analysis had two main outcomes. The first outcome had to estimate the pooled magnitude of postpartum hemorrhage in Ethiopia. Postpartum hemorrhage is defined as loss of > 500 ml of blood after vaginal delivery or > 1000 ml after cesarean delivery within 24 h. The second objective was to determine the pooled effect size of associated factors for postpartum hemorrhage. The magnitude of postpartum hemorrhage was calculated by dividing the number of mothers who had postpartum hemorrhage by the total number of mothers who have been included in the study and multiplied by 100. For the second outcome, the odds ratio was used to measure the level of association between postpartum hemorrhage and factors. The odds ratio was calculated from primary studies using two by two epidemiological tables.

### Data extraction

The data extraction format prepared in Microsoft Excel was used to extract the necessary data from each primary study. The extraction format contains the name of the first author, the publication year, the region where the studies were conducted, the sample size, the response rate, and the magnitude of postpartum hemorrhage for the first objective. For the second objective (factors associated with postpartum hemorrhage), the data extraction format was prepared in the form of two by two tables. Categorical variables (a, b, c, and d) with postpartum hemorrhage or bleeding were tabulated with outcome variables (magnitude of postpartum hemorrhage). The differences between the two authors during data extraction were solved by re-extracting the data from the primary article together. The other authors checked the accuracy of the extracted data.

### Data analysis and interpretation

The data extracted in the Microsoft Excel format was exported to Stata Version 16.0 statistical software for analysis**.** A random effect meta-analysis model was used since it considers heterogeneity among studies. In this meta-analysis, the Forest plot was used to show the pooled estimate with a 95% confidence interval (CI). Statistical heterogeneity was evaluated by the I^2^ test [34]. The heterogeneity of the included studies was interpreted as an I^2^ value of 25% = low, 50% = moderate, and 75% and above = high. In case of high heterogeneity, a subgroup was performed to identify the possible source of heterogeneity. Egger’s weighted regression test was used to assess publication bias at a 5% significance level [35]. We also assessed publication bias by visual inspection of funnel plots. The pooled effect size of factors associated with postpartum hemorrhage was estimated as an odds ratio. Finally, for all analyses, P < 0.05 was considered statistically significant.

## Results

### Search results

**Fig. 1:**
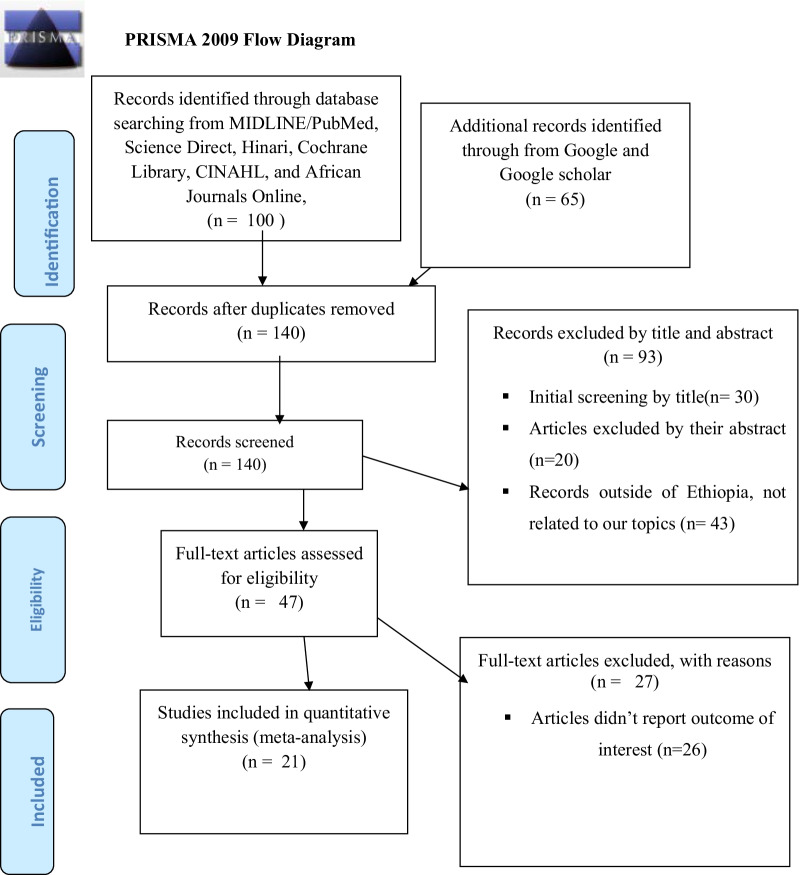
PRISMA flow chart during the selection process for the studies included in the analysis

A total of 165 records from MIDLINE/PubMed/, Science Direct, Hinari, Cochrane Library, CINAHL, and African Journals Online, Google, and Google Scholar databases were searched. Of which 25 records were excluded due to duplication. We also excluded 93 records because these articles were not related to our review after assessing their titles and abstracts. After assessing 47 full articles, 26 articles were further excluded for reasons (the outcome variables were not reported). Finally, 21 studies were included in this systematic review and meta-analysis (Fig. 1).

### Characteristics of the included articles

The current systematic review and meta-analysis included 21 primary studies with a total of 93,898 study participants. Of the total of 21 studies; 7 studies were conducted in Amhara region [11, 36–41], 6 studies in South nation nationality and people (SNNP) region [30, 42–46], 2 studies in Oromia region [10, 47], 2 studies in Addis Ababa City administration [29, 48], 1 study in Tigray region [49], 1 study in Dire Dawa City administration [50], 1 study in Harar region [51] and 1 study was conducted national-wide [52] (Table 1). Only 4 studies were conducted in rural area, the rest 17 were conducted in urban area. Among the 21 primary studies included in this meta-analysis, 19 studies were conducted in a health institution and only two studies were community-based. Three studies from 21 studies were case–control, and the rest were a cross-sectional in study design. The sample size of the primary studies included in this systematic review and meta-analysis ranged from 144 to 68,437 as reported in debre tabor Amhara region and national-wide studies respectively [39, 52]. The highest magnitude of post-partum hemorrhage was 16.6% reported in SNNP region [30], and the lowest report was 1.4% in Addis Ababa [29].

**Table 1:** Summary of the studies included in the systematic review and meta-analysis of magnitude and associated factors of post-partum hemorrhage in Ethiopia, 2021

Authors	Publication year	Region	Sample size	Magnitude (%)	Study designs	Area	Study setting	Sampling technique
Mesfin et al. [51]	2021	Harar	642	12.9	Cross-sectional	Urban	Institution-based	Simple random
Hamdela et al. [42]	2021	SNNPR	2517	2.9	Cross-sectional	Urban	Community-based	Simple random
Worku et al. [36]	2019	Amhara	384	11	Cross-sectional	Urban	Institution-based	Systematic random
Adere et al. [48]	2020	Addis Ababa	606	12.4	Case control	Urban	Institution-based	Systematic random
Abebaw et al. [37]	2020	Amhara	424	14.2	Cross-sectional	Urban	Institution-based	Systematic random
Jeilu et al. [50]	2021	Dire Dawa	239	13	Cross-sectional	Urban	Institution-based	Systematic random
Legesse et al. [10]	2017	Oromia	600	7	Case control	Urban	Institution-based	Simple random
KEBEBUSH ABERA [29]	2014	Addis Ababa	12,995	1.4	Cross-sectional	Urban	Institution-based	Consecutive sampling
Temesgen [38]	2017	Amhara	377	5.8	Cross-sectional	Urban	Institution-based	Systematic random
Kebede et al. [30]	2019	SNNPR	422	16.6	Cross-sectional	Urban	Institution-based	Consecutive sampling
TESHAGER DERBEW [45]	2017	SNNPR	1786	5.9	Cross-sectional	Rural	Institution-based	Consecutive sampling
Harrison et al. [44]	2021	SNNPR	998	2.2	Cross-sectional	Urban	Institution-based	Consecutive sampling
Abate et al. [11]	2014	Amhara	203	14.8	Cross-sectional	Urban	Institution-based	Sample random
Geleto et al. [52]	2020	National	68,437	4.2	Cross-sectional	Urban	Institution-based	Survey
Habitamu et al. [39]	2019	Amhara	144	7.6	Cross-sectional	Urban	Institution-based	Systematic random
Gudeta et al. [47]	2018	Oromia	200	9.7	Cross-sectional	Urban	Institution-based	Systematic random
Maeruf et al. [49]	2020	Tigray	752	3.1	Cross-sectional	Urban	Institution-based	Systematic random
Zimmerman et al. [43]	2019	SNNPR	321	12.5	Cross-sectional	Rural	Community-based	Survey
Tiruneh et al. [40]	2020	Amhara	1060	9	Cross-sectional	Rural	Institution-based	Systematic random
Dagne et al. [41]	2021	Amhara	493	13.6	Cross-sectional	Rural	Institution-based	Systematic random

*SNNPR* South Nation Nationality and People Region

### Meta-analysis

**Fig. 2:**
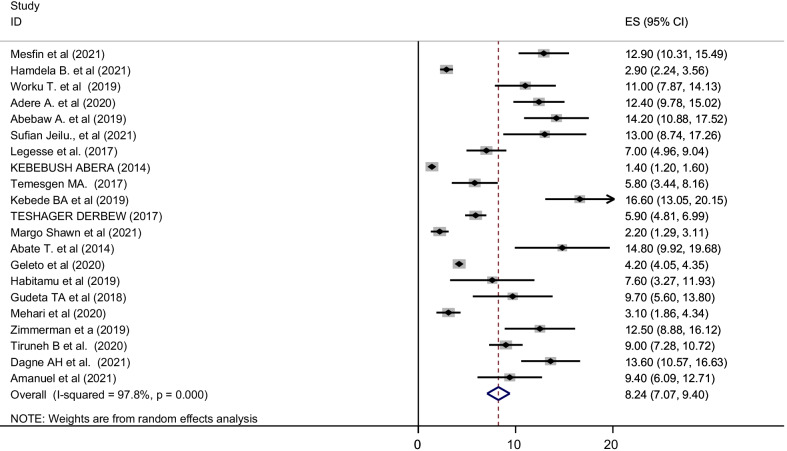
Forest plot for the pooled magnitude of postpartum hemorrhage in Ethiopia, 2021

**Fig. 3:**
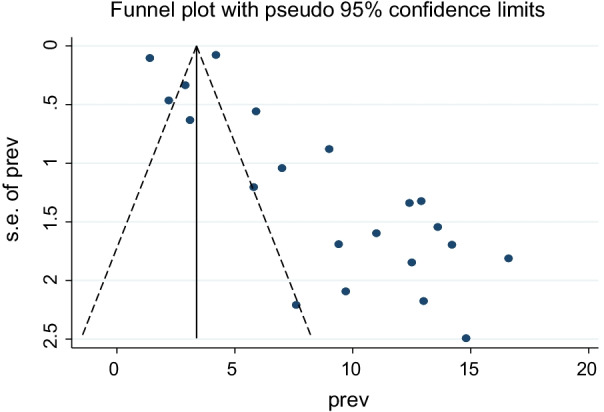
Funnel plot showing symmetric distribution of articles on magnitude of post-partum hemorrhage in Ethiopia, 2021

A random effect meta-analysis model was used to estimate the pooled magnitude of postpartum hemorrhage and associated factors in Ethiopia. The pooled magnitude of postpartum hemorrhage was 8.24% [(95% CI 7.07, 9.40), I^2^ = 97.8%, P < 0.01] (Fig. 2). Significant publication bias was observed since the Egger’s test result is statistically significant (P = 0.010) at 95% CI. The Duval and Tweedie trim and fill methods were used to estimate the number of studies missed from a meta-analysis as a source of publication bias but the finding was not significant [53]. We also observed a symmetrical distribution of the funnel plot, indicating a significant publication bias (Fig. 3).

### Subgroup analysis

**Fig. 4:**
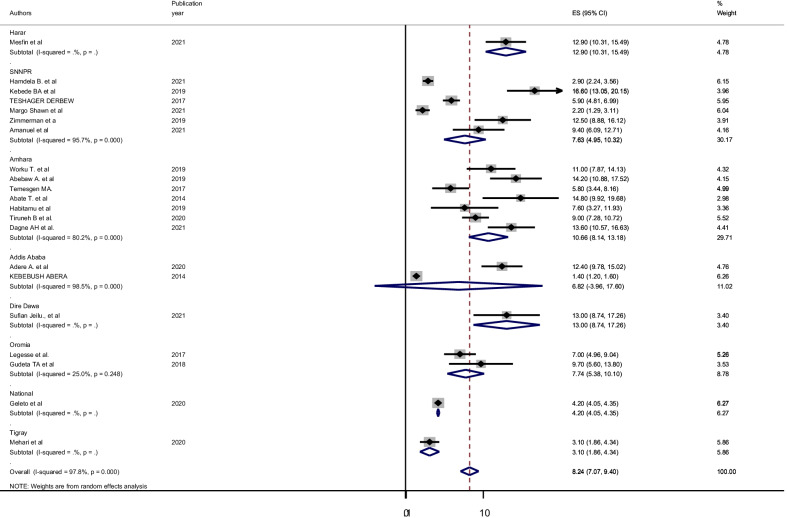
Sub-group analysis of magnitude of post-partum hemorrhage in Ethiopia by using regions, 2021

**Fig. 5:**
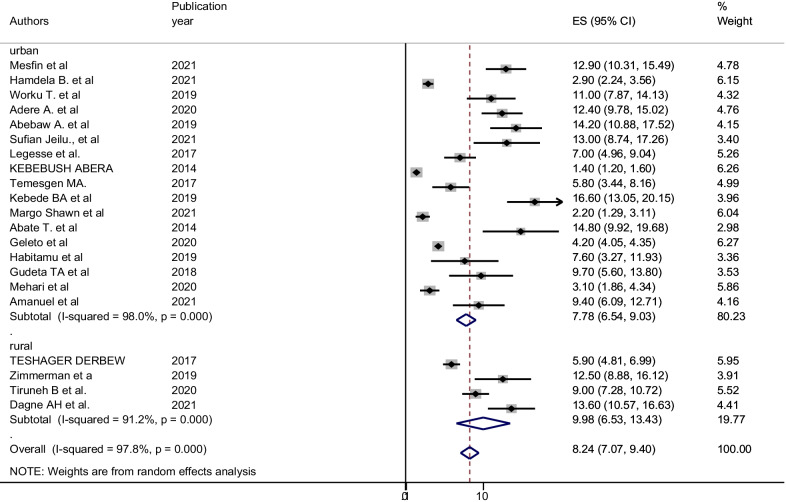
Sub-group analysis of magnitude of post-partum hemorrhage in Ethiopia by using study area, 2021

Subgroup analysis was performed to identify the source of heterogeneity since I^2^ test (I^2^ = 97.8%, P < 0.001) shows the presence of significant heterogeneity. Therefore, subgroup analysis was done using region and the study area (urban vs rural). Accordingly, the highest pooled magnitude of postpartum hemorrhage was in the Amhara region (10.66% (95% CI 8.14, 13.18), I^2^ = 80.2%) and the lowest was in Addis Ababa (6.8% (95% CI 3.96, 17.60), I^2^ = 98.5) (Fig. 4). The pooled magnitude of postpartum hemorrhage was slightly higher in rural areas 9.99% (95% CI 6.53, 13.43), I^2^ = 91.2%) than in urban areas 7.69% (95% CI 6.41, 8.96), I^2^ = 98.1%) (Fig. 5).

### Factors associated with postpartum hemorrhage

This systematic review and meta-analysis identified different factors associated with postpartum hemorrhage in Ethiopia. Variables reported had statistically significant association with the occurrence of postpartum hemorrhage in at least two primary studies were incorporated in the metal analysis. Accordingly, age of the women, prolonged labor, antenatal care (ANC) visits, parity and history of postpartum hemorrhage were significantly associated with postpartum hemorrhage (Table 2).

**Table 2:** Summary of factors associated with postpartum hemorrhage in Ethiopia, 2021

Variables	Number of studies	Studies includes	Odds ratio with 95% CI	Heterogeneity	
				(I^2^) (%)	P-value
Advanced age (≥ 35 years)	3	Kebede et al. 2019 Habitamu et al. 2019 Amanuel et al. 2021	4.61 (2.81, 7.56)	0.0	P = 0.076
Prolonged labor	3	Hamdela et al. 2021 Temesgen 2017 Amanuel et al. 2021	4.94 (2.30, 10.63)	66.0	P = 0.053
Anti-natal care follow-up	3	Temesgen 2017 Habitamu et al. 2019 Amanuel et al. 2021	8.87 (3.21, 24.48)	66.3	P = 0.051
Parity	2	Habitamu et al. 2019 Temesgen 2017	6.58 (1.90, 22.80)	58.8	P = 0.119
History of postpartum hemorrhage	3	Habitamu et al. 2019 Kebede 2019 Amanuel et al. 2021	4.12 (2.51, 6.76)	0.0	P = 0.725

**Fig. 6:**
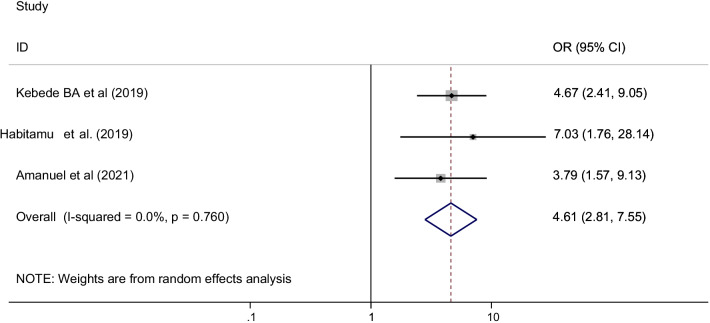
The pooled odds ratio of the association between age of the mother and post-partum hemorrhage in Ethiopia, 2021

The age of the mother was significantly associated with postpartum hemorrhage among three primary studies included in this systematic review and meta-analysis [30, 39, 46]. A total of 864 study participants were included to determine the association between the age of the mothers and the occurrence of post-partum hemorrhage. The pooled odds ratio showed that older mothers (≥ 35 years old) had 4.6 times higher risk of developing postpartum hemorrhage than mothers younger than 35 years old [OR = 4.61 (95% CI 2.81, 7.56), I^2^ = 0.0%, P = 0.0760] (Fig. 6).

**Fig. 7:**
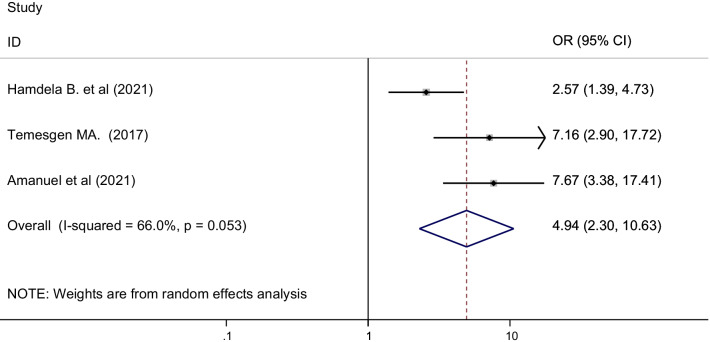
The pooled odds ratio of the association between prolonged labor and post-partum hemorrhage in Ethiopia, 2021

Prolonged labor was reported as a factor associated with postpartum hemorrhage among three primary studies [38, 42, 46]. To analyze the association between prolonged labor and postpartum hemorrhage; 1060 mothers were included in the analysis. Accordingly, the odds of postpartum hemorrhage were 5 times higher in mothers who had prolonged labor as compared to mothers whose labor had not been prolonged [OR = 4.94 (95% CI 2.30, 10.63), I^2^ = 66.0%, P = 0.053] (Fig. 7).

**Fig. 8:**
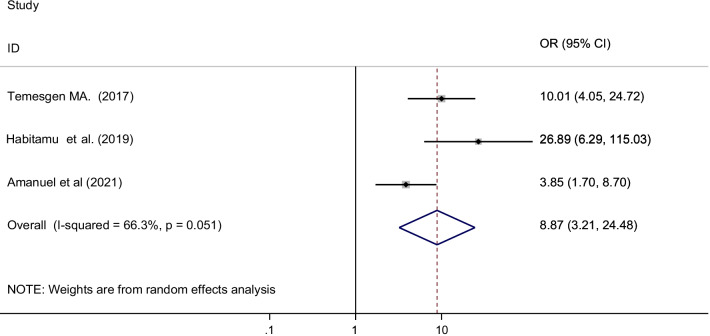
The pooled odds ratio of the association between ant-natal care (ANC) follow-up and post-partum hemorrhage in Ethiopia, 2021

Three primary studies have reported that antenatal care follow-up (ANC) was significantly reduces the occurrence of post-partum hemorrhage after birth [38, 39, 46]. More than 819 postnatal mothers were included to show the association between antenatal care follow-up and postpartum hemorrhage. The pooled result showed that mothers who didn’t had ANC follow-up were 9 times more risk to develop postpartum hemorrhage than mothers who had ANC follow-up [OR = 8.87 (95% CI 3.21, 24.48) I^2^ = 66.3%, P = 0.051] (Fig. 8).

**Fig. 9:**
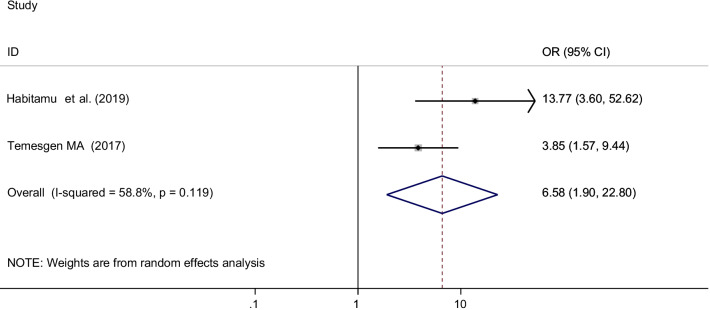
The pooled odds ratio of the association between parity and post-partum hemorrhage in Ethiopia, 2021

Parity was reported as a factor associated with postpartum hemorrhage among two primary articles included in this meta-analysis [38, 39]. A total of 521 mothers were included to analyze the association between parity and postpartum hemorrhage. The odds of postpartum hemorrhage among grand multipara mothers were 6.6 times higher than multi- and prim-Para mothers [OR = 6.58 (95% CI 1.90, 22.80) I^2^ = 58.8%, P = 0.119] (Fig. 9).

**Fig. 10:**
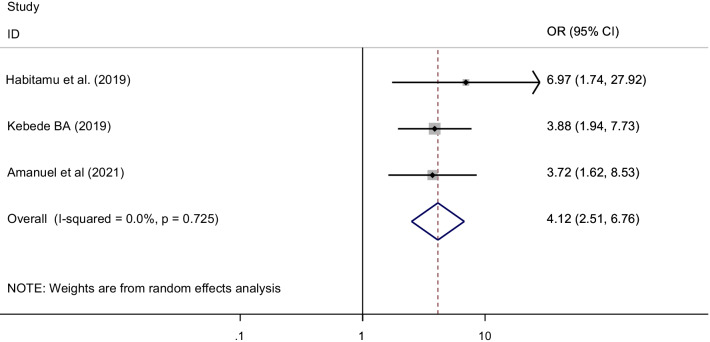
The pooled odds ratio of the association between history of previous PPH and post-partum hemorrhage in Ethiopia,2021

Three primary studies included in this meta-analysis reported that pervious postpartum hemorrhage was significantly associated with the occurrence of post-partum hemorrhage after birth [30, 39, 46]. A total of 863 mothers were included to show the association between previous PPH and postpartum hemorrhage. According to the analysis, mothers who had postpartum hemorrhage in the previous birth were 4 times higher risk of developing postpartum hemorrhage than mothers who didn’t have postpartum hemorrhage previously [OR = 4.12 (95% CI 2.51, 6.76) I^2^ = 0.0%, P = 0.725] (Fig. 10).

## Discussion

Globally, postpartum hemorrhage is a leading cause of maternal morbidity and mortality that causes more than 25% of maternal death [54] and a major cause of postpartum disability in sub-Saharan Africa [55]. Even if it is one of the leading causes of maternal mortality, most cases can be prevented through proper management, since it is manageable cause of maternal death [55]. This review was carried out to estimate the pooled magnitude and associated factors of postpartum hemorrhage in Ethiopia.

This systematic review and meta-analysis revealed that the pooled magnitude of postpartum hemorrhage in Ethiopia was 8.24%. This report was in line with studies conducted in Uganda 9% [56] and Japan 8.7% [57], but much lower than report studies in Pakistan 21.3% [58], Cameroon 23.6% [59], and Yemen 29.1% [60]. The discrepancy could be due to the difference in maternal health services utilization (prenatal, natal, and postnatal care) between the countries. The pooled magnitude of postpartum hemorrhage in this meta-analysis was greater than studies conducted in Senegal and Mali 5.4% [9], India 3.55% [61], Norway 2.5% [62] and Zimbabwe 1.6% [3]. The possible explanation for this variation could be due to geographic and sociocultural differences, as well as maternal health service utilization. The above difference might also be due to the nature of the studies between the primary studies and the meta-analysis.

This meta-analysis also determines the pooled effect of factors associated with postpartum hemorrhage among postnatal mothers. Accordingly, age of the mothers (> 35 years), duration of labor, antenatal care (ANC) follow-up, party, and history of PPH were significantly associated with postpartum hemorrhage. The odds of postpartum hemorrhage was 4.6 times higher in mothers older than 35 years as compared to the mothers younger than 35 years old. This finding is supported by study reports conducted in Uganda [56], Pakistan [58] and France [63]. The reason might be that obstetrics complications increase as the age of the mothers increases.

Mothers who had prolonged labor had 5 times higher odds of postpartum hemorrhage than their counterparts. A similar finding was reported from studies done in China [64], Pakistan [58], and Cameroon [59]. This could be due to the fact that prolonged labor causes uterine atony, which is a leading cause of postpartum hemorrhage. This meta-analysis also showed that the odds of developing postpartum hemorrhage among mothers who didn’t have ANC follow up was 9 times higher than their counterparts. This could be since mothers can obtain adequate information about institutional delivery as well as birth preparedness and complication redness during ANC visits, which can reduce the risk of postpartum hemorrhage.

This study also revealed that grand multi-Para mothers had 6.6 times higher risk of developing postpartum hemorrhage than multi-Para and prim-Para mothers. The finding is supported by studies done in Cameroon [59], Uganda [56] and Pakistan [58]. The reason might be that reduce the muscular strength of the uterus due to the loss of collagen fibres, results decreased uterine contraction after birth leads to bleeding. The odds of postpartum hemorrhage were four times higher in mothers who had history of postpartum hemorrhage than mothers who hadn’t history of postpartum hemorrhage. Similar study findings have been reported from studies conducted in Norway [62], China [64] and Cameroon [59]. The reason might be that once the mother developed PPH, the contraction of myometrium has been reduced for the next birth, which can easily develop postpartum hemorrhage. This review was used a wide range of searching strategies and two or more reviewers were involved in the whole review process through PRISMA guideline. The finding of this systematic review and meta-analysis was strongly helping different stakeholders working in maternal and child health to focus on the main contributor factors to reduce the incidence of postpartum hemorrhage. If postpartum hemorrhage is reduced, maternal death will be greatly reduced since PPH is the main cause of maternal death.

## Limitation of the study

Most primary studies included in this systematic review and meta-analysis used a cross-sectional study design, making it difficult to establish cause–effect relationships, and the outcome variable may be affected by other confounding factors. The presence of significant heterogeneity between the primary studies is the other limitation of this study.

## Conclusion

This systematic review and meta-analysis conclude that the magnitude of postpartum hemorrhage in Ethiopia was moderately high. Advanced age, prolonged labor, absence of ANC visit, grand multiparty, and history of postpartum hemorrhage were identified as associated factors for the occurrence post-partum hemorrhage. Health professionals attending labor and delivery should give more attention for high-risk mothers (older age, grand-multi parity and history of PPH) mothers during delivery. Continue to encourage ANC visit and prevent prolonged labor should be recommended to reduce the occurrence of postpartum hemorrhage.

## Supplementary Information

**Additional file 1.** PRISMA 2020 Checklist.

## Data Availability

The data set analysed during the current study is available from the corresponding author on reasonable request.

## Abbreviations

ANC: Antenatal care
MMR: Maternal mortality rate
PPH: Post-partum hemorrhage
WHO: World health organization
SEA: South East Asian
SSA: Sub-Saharan African
EDHS: Ethiopia demographic health survey
CSA: Central statistical agency
PRISMA: Preferred reporting items for systematic reviews and meta-analysis
ARDS: Acute respiratory distress syndrome
ARF: Acute renal failure
